# Translation and cross-cultural adaptation of the young children participation and environment measure for its use in Austria, Germany, and Switzerland

**DOI:** 10.3389/fped.2023.1258377

**Published:** 2024-01-04

**Authors:** Beate Krieger, Friedrich Ederer, Ruth Amann, Thomas Morgenthaler, Christina Schulze, Britta Dawal

**Affiliations:** ^1^ Institute of Occupational Therapy, School of Health Sciences, Zurich University of Applied Sciences, Winterthur, Switzerland; ^2^ Department of Economic and Social Sciences, Institute for Social Medicine, Rehabilitation Sciences and Health Services Research, Nordhausen University of Applied Sciences, Nordhausen, Germany; ^3^ Department of Occupational Therapy, Graz University Clinic for Pediatrics and Adolescents Medicine, Graz, Austria; ^4^ Department of Education and Social Sciences, South Westphalia University of Applied Sciences, Soest, Germany

**Keywords:** assessment, children, social participation, environment, home, school, community, think-aloud

## Abstract

**Background:**

Concepts such as participation and environment may differ across cultures. Consequently, cultural equivalence must be assured when using a measure like the Young Children Participation and Environment Measure (YC-PEM) in other settings than the original English-speaking contexts. This study aimed to cross-culturally translate and adapt the YC-PEM into German as it is used in Germany, Austria, and Switzerland.

**Methods:**

Following international guidelines, two translations were compared, and the research and expert team made the first adaptations. Twelve caregivers of children with and without disabilities from three German-speaking countries participated in two rounds of think-aloud interviews. Data were analyzed by content analysis to look for item, semantic, operational, conceptual, and measurement equivalence to reach a cultural equivalence version in German.

**Results:**

Adaptations were needed in all fields but prominently in item, operational, and conceptual equivalence. Operational equivalence resulted in graphical adaptations in the instructions and questions to make the German version of YC-PEM, YC-PEM (G), more user-friendly.

**Conclusion:**

This study presents a cross-cultural translation and adaptation process to develop a German version of the YC-PEM suitable for Germany, Austria, and Switzerland. A culturally adapted YC-PEM (G) is now available for research, practice, and further validation.

## 1 Introduction

“Participation” and “environment” are concepts that are meaningful for a person's life even at a very young age. The World Health Organization introduced within the international classification of function, disability, and health (ICF) (1, 2) these two concepts and defines participation as “*involvement in life situations”* and environment as “*the physical, social, and attitudinal environment in which people live and conduct their lives*”. The model of the family of participation-related constructs (3) provides a more precise definition, in which participation is two-fold: attendance and involvement. Attendance describes someone being present in a life situation while involvement describes the personal experience during this life situation. According to these authors (3), attendance and involvement are influenced by three related concepts; preferences (the opportunity to choose and undertake activities), activity competence (the ability to execute the activity according to a standard), and sense of self (participation related to confidence, satisfaction, and self-esteem). While these aspects are distinct from participation, they are critical to any participation experience. Furthermore, all mentioned aspects are dependent on external factors including the environment which determines how available, accessible, affordable, accommodating, and acceptable opportunities are (4, 5).

The focus on participation and environment has slowly but fundamentally shifted researchers' views on disability, interventions, and the use of rehabilitation outcome measures for all age groups (6–9). Yet, for the practice of childhood rehabilitation, a paradigm shift is demanded to guide the knowledge transfer from research into participation-based practices (10). Seen from a family-system view (11), young children, caregivers, and environments are strongly intertwined. While the interconnection between participation and environment is seen as central for further interventions in children and adolescents (12, 13), these concepts are rarely explored and assessed together in young children between 0 and 5 years of age.

Young children participate when they interact with others during personal care, play alone or with others, or attend family gatherings in the community or at other peoples’ places (2). Young children's environments are fundamentally shaped by those who take care of them.

Parents, subsequently called “caregivers”, provide emotional and physical care for their children at home, which is influenced by caregivers' choices, values, and socio-economic possibilities. Similarly, caregivers' choice of childcare and preferences for community places shape young children's participation (14) and vice versa (15). Other environmental aspects are inherently framed by macrosystems such as local or governmental policy and regulatory bodies (16). Examples are how maternity leave is organized after childbirth or what policies influence playground design and safety. Compared to older children, young children are more dependent on existing environments as they are less able to influence environments themselves (17, 18). Systematic reviews and empirical research have found that environmental factors mediate the participation of children at home, pre-school or school, and in the community to a high extent (12, 14). Therefore, it is of practical importance to assess both the participation and environment of young children under the age of 5.

For German-speaking countries, no such measurement is, to the best of our knowledge, available. German is mainly spoken in Central Europe and is the native language of almost 100 million people. It is the official or co-official language in Germany, Austria, Switzerland, Lichtenstein, and in the German-speaking communities in South Tyrol (Italy), Belgium, Luxembourg, and Poland. However, different regions use different dialects: words or phrases used in one country are not understood or differ in meaning in another country. As health professionals in these countries work closely together and worker mobility is high, joint assessments are needed. Therefore, we intended to find a measurement that assesses participation and environments in young children and is suitable in all German-speaking countries.

The Young Children Participation and Environment Measure (YC-PEM) is a 28-item assessment modeled after the Participation and Environment Measure of Child and Youth (PEM-CY) (19). It was developed to provide a comprehensive, detailed, and feasible tool as a proxy assessment of young children between 0 and 5 years of age (20). Caregivers rate in three settings (home, day care/preschool, and community) activities in which their children participate and the environmental features of these settings. Caregivers are asked to rate the activities in which their child participates, as well as the environmental features across these three settings. For each activity type, caregivers are prompted to assess their children's participation along three dimensions. Firstly, they evaluate attendance using an 8-point scale ranging from “never” (0) to “once or more each day” (7). Secondly, they rate their children's level of involvement on a 5-point scale from “not very involved” (1) to “very involved” (5). Thirdly, caregivers indicate whether they desire a change in their children's participation, selecting “yes” (1) or “no” (0). If a change is desired, caregivers specify the preferred direction of change: “more or less frequent participation,” “more or less involved participation,” or “more engagement in other activities.” Additionally, caregivers are prompted to describe strategies they have already applied to enhance their children's participation.

Following this, caregivers evaluate the impact of environmental features and resources on their children's participation in each setting. This includes assessing 13 items at home, 16 items for daycare/preschool, and 17 items for the community. Examples of these items include the physical layout of the home, the attitudes and actions of personnel at daycare, and the safety of the community.

Psychometric properties of YC-PEM were examined with 395 children (302 without disability and 93 with developmental disability) (20). The internal consistency ranged from .68 to .96 in the participation scales and from .91 to .96 in the environment scales. Test-retest reliability after 4 weeks ranged from .31 to .93 in the participation scales and from .92 to .94 in the environment scales. One of the three participation scales and the environment scales demonstrated significant group differences by disability status across all three settings, and all four scales differentiated disability groups in the daycare/preschool setting. The environmental scales of YC-PEM showed adequate concurrent validity with the Craig Hospital Inventory of Environmental Factors—Child and Parent Version (CHIEF-CP) (21).

Since its introduction, the YC-PEM has been used for care planning (22), description of disability-specific participation and environment patterns (23, 24), and assessments of changes over time (25). Recent research has established an electronic-based version (YC-PEMe-PRO) (26) based on qualitative interviews with caregivers (27). YC-PEM was developed according to a similar assessment, Participation and Environment Measure—Child and Youth Version (PEM-CY) (19) which covers children and adolescents from 5 to 17 years of age.

Within the global use of ICF concepts, different cultures and societies shape how “participation” and “environment” are experienced concretely in real life. As such, related assessments should be used universally (28) and at the same time reflect cultural values. Therefore, equivalence, specifically conceptual equivalence, is essential because concepts such as participation and disability may differ across cultures (29). Based on experiences of translation and cultural adaptation of YC-PEM to the context of Singapore (30), Sweden (31), China (32), Dutch-speaking countries (33), and Hispanic US population (34), a guiding process to culturally adapt participation-focused pediatric practice was recently published (35). According to these authors, some of which are the developers of the YC-PEM, cross-cultural research advances the delivery of culturally responsive pediatric rehabilitation and translates knowledge on a global scale. They argue that to effectively capture the complex concepts of participation and environments, valid, reliable, and culturally sensitive measures are needed. In accordance with other authors (3, 17, 36), they further argue that practitioners can only develop meaningful and client-driven participation goals in this way and subsequently evaluate these in accordance with their cultural context. These participation and environmental-focused assessments developed and used primarily in North America might not be culturally suitable in other countries and cultural contexts (37, 38) such as in the German-speaking countries.

The aim of this study is to cross-culturally translate and adapt the YC-PEM into German as spoken in three German-speaking countries (Germany, Austria, and Switzerland).

## 2 Methods

### 2.1 Design

Cross-cultural adaptation “encompasses a process which looks at both language (translation) and cultural adaptation issues in the process of preparing a questionnaire for use in another setting” (39). We adhered to long-established guidelines (40) in combination with six categories of equivalence (29) which were adapted and described by Stevelink and von Brakel (41) specifically for participation assessments (p. 1257) (see Table 1).

**Table 1:** Definition of equivalence adapted from Stevelink and van Brakel (41).

Equivalence	Definition
Item	Exists when items estimate the same parameters and are equally relevant in both cultures.
Semantic	Reached when the transfer of meaning across languages is achieved.
Operational	Operational equivalence is the possibility of using a similar question format, instructions, mode of administration, and measurement method. Thus, the psychometric properties of the adapted versions are equivalent.
Conceptual	Achieved when the measurement has the same relationship to the underlying concept in both cultures, specifically in the domains included and the emphasis placed on different domains.
Measurement	The psychometric properties of the adapted version of the participation measures are equivalent.
Cultural	A summary of the five other equivalences: the extent to which an instrument is equally suitable for use in two or more cultures.

### 2.2 Settings of early childcare in three German-speaking countries

Germany, Austria, and Switzerland are independent national entities with state-supported health care and federally organized educational systems. All three countries support families with small children and provide financial support, early counseling support (such as midwifery and early educators), and early medical treatment. While early childcare possibilities are not obligatory, children in all three countries attend kindergarten before entering school. As the Salamanca Declaration (42) recommended, all three countries aim to include children with special needs in mainstream schools. However, due to the different national political systems, there are essential differences in regulations influencing early childcare of families, preschool, and health services. Maternity leave after childbirth varies between 8 weeks (G) and two years (AU). After caring for small children for 3 years, parents in Germany are legally entitled to an equivalent type of job, which means that German parents often stay longer with with their young children at home. Preschool or kindergarten is not compulsory before four years of age (Switzerland) and 6 years of age (Germany). The models of state-run and private preschool institutions vary greatly, reflected by models, names, and how they are financed. Linguistic examples are “nurseries”, “kindergartens”, “pre-schools”, “play groups”, “forest groups”, or “foster families”. While medical treatment is financed in Austria and Germany through the health insurance of caregivers, children in Switzerland have their own health insurance, and caregivers must pay 10% of all ambulatory costs. Thus, to summarize, a cultural adaption of the YC-PEM into German is needed to integrate a second cultural adaption into three similar but also different national contexts.

### 2.3 Research group, experts, developers, translators, and caregivers

**Figure 1:**
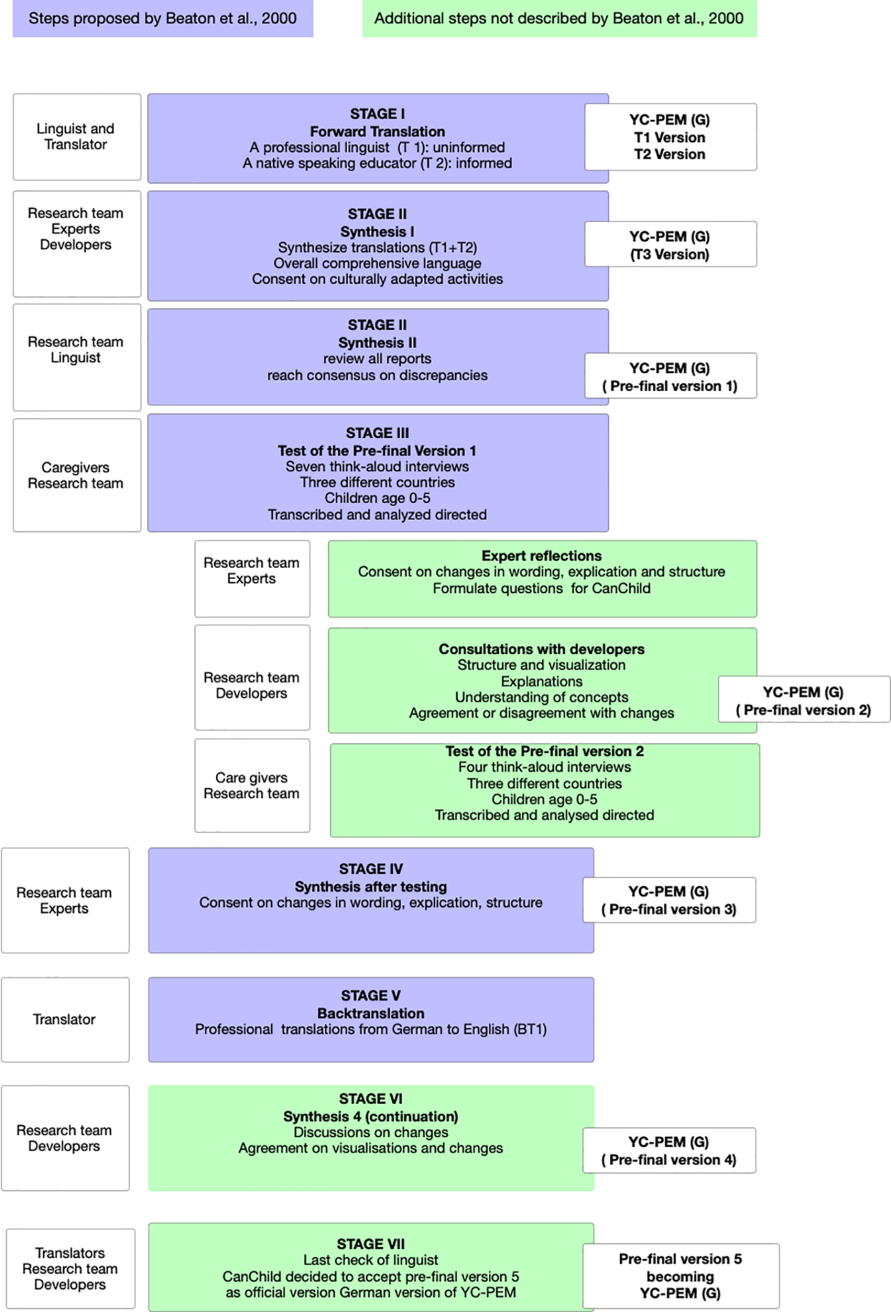
Process of cross-cultural adaptation of YC-PEM to YC-PEM (G).

During different stages of this research, a variety of persons participated. (1) The research team (BK, FE, CS, RA, TM, and BD) consisted of professionals with experiences in childhood practice and research from the three German-speaking countries. (2) The expert committee reflected a multi-perspective view on the life of young children in the three countries. It consisted of parents of young children, occupational therapists, early pedagogical educators, health care methodologists, and early childhood researchers. (3) The developers represented by the YC-PEM team of CanChild were consulted and informed regularly after signing a translation agreement. (4) A professional linguist and a native-speaking teacher oversaw the translations and spellings. (5) Twelve caregivers of children with and without disabilities participated in qualitative think-aloud interviews. Criteria for inclusion were: (a) caring for a child between 0 and 5 years of age, (b) living in Germany, Austria, or Switzerland in the last five years, and (c) being able to read and speak German. As reported in similar work (31, 33, 34), convenience sampling was used to recruit caregivers. This was combined with a purposeful sampling technique to reach diversity. Sampling was conducted in two rounds (see Figure 1). All participants consented verbally and in written form to their own participation and on behalf of their children. Table 2 describes the characteristics of the sample. Ethical approval was obtained in all countries from regional ethical committees: the Ärztekammer Westfalen-Lippe und WWU Münster (Germany: 2020, February 14; Vg-Nr. 51451510), Kantonale Ethikkommision Zürich (Switzerland: 2020, February 6; BASEC-Nr. REQ-2020-00114), and Ethikkommision der Medizinischen Universität Graz (Austria: 2020, June 10; EK-Nr: 32-243 ex 19/20).

**Table 2:** Participant characteristics per interview round and country.

County	Round	Characteristics of caregivers							Characteristics of children		
		Pseudonym	AiY	Family status	Level of education	Job title	Work load	Living context	AiM	Sex	Medical diagnosis
Germany	1	Ann	29	Couple	High	Science assistance	Reduced	Sub urban	51	Male	Expressive Language Disorder
	1	Christine	30	Couple	Middle	Manager	Not working	Sub urban	14	Male	n/a
	2	Jane	42	Single	Middle	Housewife	Looking for work	Rural	3	Female	Trisomy 18
	2	Jim	32	Couple	Low	Gardener	Fulltime	Inner city	11	Male	Spina bifida
Austria	1	Kim	41	Couple	Middle	Lecturer	Reduced	Inner city	28	Female	Trisomy 21
	1	Julia	28	Couple	High	n/a	Looking for work	Inner city	58	Male	Developmental disorder
	1	mother	28	Couple	High	Speech therapist	Other	Inner city	3	Male	n/a
	2	Mary	35	Couple	Low	Foster mother	Reduced	Rural	19	Female	Disorder autonomic nerve system
Switzerland	1	Sofia	38	Couple	High	Business continuity	Reduced	Sub urban	24	Female	n/a
	1	Naomi	40	Couple	High	Midwifery	Reduced	Inner city	48	Female	n/a
	1	Sarah	45	Couple	Middle	Physio-therapist	Looking for work	Sub urban	60	Male	Autism spectrum disorder
	2	Veronica	35	Couple	High	Administration	Reduced	Rural	9	Female	Cerebral palsy

AiY, age in years; AiM, age in months; ASD, Autism spectrum disorder.

### 2.4 Procedures

• *Stage I: Forward Translation:* Adhering to the guidelines (40), we started with two independent forward translations of the English YC-PEM into German (T1 and T2). To keep consistency with already translated and adapted version PEM-CY (G) (43), equal conceptional words (e.g., “participation” (German: “Teilhabe”), “community” (German: “Gesellschaft”), or “involvement” (German: “Engagement”)) were used.
• *Stage II: Synthesis 1:* The research team compared the two translations and developed one synthesized version (T3). The focus was on ensuring comprehension of the wording in all three countries. The team of authors decided against a back translation at this stage because it expected a lot of adaptations as reported elsewhere (31, 43). Next, the experts and research team identified activities and expressions that required cultural adaptation and discussed them until a consensus was reached. In between, unresolved differences were discussed with the developers from CanChild, who provided advice on the discrepancies. In the second phase of synthesis II, the research team and the linguist ensured comprehension and readability, which led to the first German version of YC-PEM called YC-PEM (G) (Pre-final Version 1).
• *Stage III: Tests of the Pre-final Versions:* The pre-final Version 1 was tested with concurrent think-aloud interviews (44, 45) using a 3-step procedure (46) with eight caregivers of young children from three countries. The participants filled out a paper version of the questionnaire while the researchers observed them and followed their expressed thoughts by picking up on them and addressing them. This was followed by questions about the text. The recommended adaptations were then discussed. This process allowed the researchers to assess unique higher-level thought processes while identifying individual differences in task performance (47). A pre-tested, semi-standardized interview guide and personal briefing of interviewers who were all part of the research team ensured a similar process in all three countries. Once themes identifying the need for adaptations emerged recurrently, the research team stopped the first round and started the analysis (see the steps below for details). Parents' performance and suggestions for improvements were the focus of the analysis and were discussed with the experts. The adaptations that the participants agreed on were next presented to the developers. After an iterative agreement, the next version (Pre-Final Version 2) was then used in a second round of interviews with four other caregivers until no new or unsolved issues emerged.
  • *Stage IV: Synthesis of testing:* Following the research team's analysis, the expert committee discussed the remaining issues of round 2 that led to Pre-final Version 3.
  • *Stage V: Back translation:* A professional translator blinded to the original version translated the Pre-final Version 3 into English (BT1).
  • *Stage VI: Synthesis 4:* After presenting and explaining all differences between the original version and the German Version (Pre-final Version 3) to the developers, an iterative process led to the finally consented version (Pre-final version 4).
  • *Stage VII: Final agreement:* The linguist checked the text for comprehension, spelling, and grammar and made minor revisions (Pre-final Version 5), leading to the final version of the YC-PEM (G).

### 2.5 Analysis

The analysis aimed to examine the translation and cultural adaptation of the YC-PEM to reach the six equivalences described by Stevelink and van Brakel (41). The research team precisely documented all adaptations during the five stages, including when the need for adaptations emerged and on what evidence they were grounded.

We estimated the number of items adapted for an item and semantic equivalence by applying a summative content analysis (48). Additionally, the researchers investigated how caregivers, experts, and developers from CanChild reasoned for these adaptations.

The research team judged operational equivalence by analyzing the comments of caregivers on the adaptations concerning format, instructions, and mode of administration.

Next, for conceptual equivalence, the researcher used the concepts elaborated during the adaptation of the PEM-CY (G) (43) and integrated them into the YC-PEM translation. To verify this procedure, prompting questions were incorporated into the think-aloud interviews with caregivers, such as “*How do you understand the word “involvement” (German: Engagement)?”* The parts of the interviews relating to the assessment were transcribed. When the parents described their children's participation, the researchers summarized the content based on the decisions of two people. Next, the researchers analyzed all scripts using a direct content analysis (48). The direct content analysis allowed qualitative deepening questions such as “How were the caregivers’ conceptualizations of participation assessed?” “Were any theoretical arguments presented to question or reject conceptual equivalence?” and “How was the appropriateness of the instrument judged for use in the study population?” (41) The research did not analyze measurement equivalence, and we also refrained from additional testing. Finally, to estimate the overall cultural equivalences, the research team discussed the summarized results presented as a consensus version in the following results. Qualitative data were analyzed in German and translated into English at the very last step of analysis. This approach prevents out-of-context interpretation (49).

## 3 Results

The translation and cultural adaptation of the YC-PEM to the YC-PEM (G) has been successfully completed. The comparison of the original YC-PEM and the approved back-translated version (Pre-final version 5) showed that most of the instructions and items in all sections of the YC-PEM needed revisions after substantial feedback from caregivers, the expert committee, and the research team. After presenting the results of overall adaptations made, we delineate the results by the six equivalent adaptation categories.

### 3.1 Adaptations

Two hundred and twenty-five adaptations were made to cross-cultural translate and adapt the YC-PEM (G) from YC-PEM. During translations and the synthesis process in combination with research and expert teams, 56% of the adaptions, mostly changing of words, were made. Analysis of the interviews resulted in more frequent adaptions in instructions, visualization, and structure (see Table 3). The distribution over the three parts of the YC-PEM was almost even. Table 4 lists the adaptations and coherent equivalences in a timeline throughout the five stages of the research process.

**Table 3:** Frequencies of adaptations per stage and type.

Type of adaptations	Stages I–II	Stages III–IV	Stage V–VII
	Preparing for interviews	During interview phases	After interview phase
*N* = 225	*n* = 148 (56%)	*n* = 52 (23%)	*n* = 25 (11%)
Words (without word units^a^)	140 (62%)	26 (11%)	22 (10%)
Instructions	8 (4%)	19 (8%)	3 (1%)
Visualizations	0 (0%)	4 (3%)	0 (0%)
Structure	0 (0%)	3 (1%)	0 (0%)

^a^ Word units are composed of some words to express one meaning (see semantic equivalence).

**Table 4:** Development of equivalence or put it in the sub-element.

		Original	Type of cultural adaptation					Translations and cultural adaptations
Stage	Version YC-PEM	YC-PEM (English)	Item	Semantic	Operational	Conceptual	Measurement	YC-PEM (G) (German)
STAGE I	Translation (T1/T2)	• “Participation” “involvement” • “Environment” • “Community” • “Activities”				x		• Use the same conceptual translation as in PEM-CY(G) (Krieger et al., 2020) to keep the conceptual connection between both questionnaires
		• Wording (“he or she”)		x				• Use of the German neutral form it (G: “es) as article for the word “child”
		• Wording (“basic care routine”)	x					• There are many options to translate into German” Grundfürsorge”, “alltägliche Verrichtungen”, “alltägliche Routine” It was included in the interviews
		• Wording (e.g. “mealtime”)		x				• Avoid country-specific wordings e.g. “Essenszeiten” (Austria) versus “Mahlzeit”(Germany), “Znüni”(Switzerland)
STAGE II	Synthesis (T3)	• Wording (e.g. “daycare/preschool”, “circle time”)		x				• Agreement to choose words commonly used in all three countries e.g. (G: “ausserfamiliäre Betreuungsangebote”, “Morgenkreis”)
		• Wording (“getting rest”, “cleaning up”)	x			x		• Widening meaning to capture cultural relevance for children e.g “rest and sleep” (G: Ruhe und Schlaf) or “tidying and cleaning” (G: “aufräumen und sauber machen”)
		• “Young children”		x				• Agreement on young children (G:“junge Kinder”) instead of (G) “Klein und Vorschulkinder” to keep the age range and a simple expression
		• “Socializing with friends”				x		• Replacing “friends” with “other children” (G: “Zusammensein mit anderen Kindern”) as friends in German is a more closer relationship, also for young children
		• Activities	x					• Change activities: - Removal of activities that are not typical in German speaking countries for young children (e.g., “yard work”, “workbook”, “parent night out”, “participate in fundraiser”, “school concert”)
				x				Present activities instead of things e.g. “siblings” changed to “take care of siblings” (G: sich um Geschwister kümmern)
			x					- Addition of culturally adapted activities, e.g. “showering” (G: Duschen”) “cutting, chopping, mixing” (G:”schneiden, zerkleinern, vermengen”) “, “zoo” (G: “Zoo”), farm (G: “Bauernhof”), “theater” (G: “Theater”), “museum” (G: “Museum”) “forest” (G: Wald”) “bicycle with child seat or trailer” (G:”Fahrrad mit Kindersitz oder Anhänger”)
		• General wording (e.g. “things at home”, “light”, “clima”)	x					• Small changes in used language structure, e.g. “aspects at home” (G: “Aspekte”), “lighting conditions” (G: “Lichtverhältnisse”), “seasons” (G: Jahreszeiten”)
		• Umbrella term (e.g “arts, craft, stories, music”; “recreational activities and trips”)		x				• Use more suitable umbrella terms “Creative” (G: “Kreatives”) and “unstructured activities and trips” (G: “unstrukturierte Aktivitäten und Ausflüge”)
STAGE III	Pre-final 1	• Adaptations in the introduction:						• Adaptations in the introduction:
		- “Health”	x			x		- Remove referring to “health”. Instead using “well-being” (G: Wohlbefinden”)
		- Prompt character			x			- Use more strict wording to guide caregivers more (G: “immer”, “relevant”)
		- Presentation as text			x			- Insertion of two pages with graphical presentations to support understanding of the process of filling the survey correctly.
		- “Participation in types of activities”		x				- Deleting “types”: participation in activities (G: Teilhabe an Aktivitäten”
		- “4 months”			x			- Highlight 4 months frame
		• Involvement: “no matter how much assistance or adaptation is used.”				x		• Add a sentence that this survey is not about the child’s independence (G: … unabhängig davon, wie selbständig ihr Kind ist”)
		• Explanation daycare section			x		x	• Add a new question for children not attending any daycare: “ my child has made use of any daycare the last four months “ (G: Mein Kind hat in den letzten 4 Monaten ausserfamiliäre Betreuungsangebote angenommen”) If the answer is “no, skip this section and continue with community participation” (G: Nein: diesen Abschnitt überspringen und bei der gesellschaftlichen Teilhabe fortfahren”)
		• Structure of questions: - “ Do the following things in your home environment help or make it harder for your child to participate in these activities at home?”		x		x		• Questions structure changed - “What influence do environmental aspects at home have on your child’s participation in all of the activities mentioned so far? (G: “Welchen Einfluss haben die Umfeldaspekte zu Hause auf die Teilhabe Ihres Kindes an allen bisher genannten Aktivitäten?”)
		- “Are the following available and/or adequate to support your child’s participation at home?”		x				- “Do you need the following aspects to support your child’ as participation at home? If yes, are they available/adequate?” (G: “Benötigen Sie folgende Aspekte zur Unterstützung der Teilhabe Ihres Kindes zu Hause? Wenn ja, sind sie vorhanden/ ausreichend?”)
		- “If you selected YES to Question C, please describe up to three strategies that you have tried to help your child participate successfully in this type of activity. If you responded “no change desired” to all of the questions above, please proceed to the next page.”				x		• Ask for strategies regardless of the given answers: - “Please describe up to three strategies you have used to try to help your child participate successfully in these activities.” (G: “Bitte beschreiben Sie bis zu drei Strategien, mit denen Sie und andere Personen versucht haben, Ihrem Kind zu helfen, sich erfolgreich an diese Aktivitäten zu beteiligen.”)
		• Wording (e.g. “strength, endurance, coordination”)		x				• Replace single words into activities of young children e.g. strength when crawling, endurance when running, skill when doing handicrafts” (G: Kraft beim Krabbeln und Klettern, Ausdauer beim Rennen, Geschicklichkeit beim Basterln”)
		• Changes in wording:						• Changes in wording:
		- “Texture of objects”	x					- “Texture of surfaces” (G: “Oberflächenbeschaffenheit”)
		- “Policies”		x				- “Rules and regulations” (G: “Gesetze und Regelungen”)
		- “Babysitters, therapists, and other professionals who care for your child at home”	x					- Shortening person as they are too many different: “…those who care for your child at home” (G: Personen, die sich zu Hause um Ihr Kind kümmern”)
		- Sequence: “Getting rest”		x				- Starting with an obvious “mealtime” instead of “rest”. Switch of positions of items
	Pre-final 2	“Daycare environments”	x					• Added names of “daycare environments (in facilities such as crèche, day-care center, kinder garden, etc. or with childminders) “ (G: “Umfeld der ausserfamiliären Betreeungsangebote (in Einrichtungen wie z.B. Krippe, Kindertagesstätte, Kita, etc. oder bei Tageseltern) “
		“Your child’s relationship with peers”				x		• We added examples e.g. “Making and maintaining friendships” (G: Freundschaften schliessen und pflegen”)
		“Social gatherings”				x		• Clarification by “social gatherings in public spaces”, (G:” Soziale Kontakte im öffentlichen Raum”)
		Response item (e.g. “once in the last month”, “no change required”)	x					• Revised to make it more explicit e.g. “once a month” (G: “einmal im Monat”), “no, it is ok as it is” (G. Nein, es ist ok so”)
STAGE IV	Pre-final 3	• Response items e.g. “no impact”		x		x		• Change answer to make it more explicit: “are currently not an issue for us” (G: “sind derzeit kein Thema für uns”)
		• Structure: subtitles			x			• Add subtitles to A (frequency), B (involvement), C (wished changes)
		• Parental answers				x		• Add line for comments (voluntary) for all activity groups
STAGE V-	Pre-final 4	• “Basic care”	x			x		• Change of heading “Daily routines for care and wellbeing”; (G: “Tägliche Routinen der Körperpflege und des Wohlbefindens»
		• “Travel outside your community”	x					• Remove community: “trips with vacations and visits” (G: “Ferien und Besuche”)

### 3.2 Item equivalence

Out of 188 adaptations, most differences emerged in “activities” (*n* = 108). They comprised 17 umbrella terms, such as “recreational activities and trips,” which we changed to “unstructured activities and trips.” For German speakers, structured activities can also be recreational. To adhere to the following examples, such as playgrounds and parks, beaches, hiking, bikes, and scooters, “unstructured” was better. The remaining adaptations to reach item equivalence comprised 91 changed activities. While some are unknown in German-speaking countries (e.g., “parents night out” and “participating in fundraising”), others were, according to parental feedback, not used with babies and toddlers (e.g., “soccer,” “T-ball,” “gymnastics,” “dance,” and “martial arts”). Replaced items reflect activities of young children in German-speaking countries (e.g., “showering,” “cutting, chopping and mixing,” “baby sport,” “zoo,” “farm,” “forest,” and “bicycle with child seat or trailer”). The researcher also discussed activities such as “sledding,” “climbing,” or “playing at the beach,” which were equally typical in all three German-speaking countries. Lastly, the word “thing,” often used in the original YC-PEM in phrases such as “things at home,” were negatively commented on by caregivers. “*We do not only modify “things”, we change our behavior, our feedback, our planning to help our child to participate more actively”* (Ann, Germany, 1.round). Caregivers felt “aspects” are a better word to ask for all kinds of strategies they use to support their children. Lastly, for item equivalence, the research team refrained from the proposed wording that reflect the language of one single German-speaking country. Examples are “Kindermädchen”, (we used “Babysitter”) or “Tierpark” (we used “Zoo”).

### 3.3 Semantic equivalence

During the process, 50 words were adapted, added, or removed to ensure a semantic equivalence for German as understood in Austria, Germany, and Switzerland.

Specifically, many semantic adaptations were needed in the three sections of the environment. One example is the factors asking about the physical, cognitive, and social demands of typical activities (items. 3–5). Caregivers struggled to transfer the abstract terms of physical demands (strength, endurance, and coordination) to concrete activities. Therefore, the research team added examples of activities associated with these demands in all sections. For example, referring to physical demands at home, “e.g., strength when crawling and climbing, endurance when running, and skill when doing handicrafts,” were added. Cognitive demands of daycare/preschool are another example: these were explained with “e.g., paying attention in a circle, learning numbers, and problem-solving when building and putting things together.” In the community environment, the social demands were explained with: “e.g., saying hello to neighbors and asking for buns in the bakery.” These adaptations were shown to be applicable to the caregivers, as less feedback was given regarding these environmental factors in the second round of interviews.

Next, participants expressed enormous difficulties with the answer option “no impact” within the response items of all environment sections. A mother reflected*: “I am perplexed. Does it mean a supportive environment exists and, therefore, the environment has no impact? Or does it mean you don’t realize any impact of the environment? We deliberately chose a child-friendly living area, so it is normal for us to live in a child-adapted environment. So, what to choose: no impact or huge impact?” (Sofia, Switzerland, 1. round)*. To clarify, we changed this answer option to “Are currently not issues for us.”

Lastly, umbrella terms helped to structure the YC-PEM. However, it was even astonishing that caregivers did not recognize existing umbrella terms in the first round. One participant said: “*These are a lot of interesting activities, but can they not group it into 4–5 they put together? It is exhausting to see so many papers and imagine you are going to fill them all out” [sic] (Julia, Austria, 1.round).* Analyzing similar statements, the researchers explained the confusion in how languages express umbrella terms. In German, they never repeat subterms. So instead of using “arts, craft, stories, and music”, we chose “creatives” for the German version as “art” in German is quasi-exclusively used for adults. On the other hand, the English umbrella term “social gathering” in the community sections needed to be translated with “social gatherings in public spaces” to be understood by German-speaking caregivers.

### 3.4 Operational equivalence

Caregivers strongly commented on operational adaptations during both interviews. Specifically, reading the instructions without graphical support (first interview) was hard for many participants. For example, one caregiver stated: “*I think this is, really, much too much. I feel like in an exam, trying to sort my thought out but always being confronted with new aspects…..even the spacing is too narrow, it is hard to stay even in one line”* [sic] (Christine, Germany, 1. Round). Others just skipped the introduction commenting on reading it later when they understood the questionnaire better. Most caregivers asked for a graphical representation, “*similar to when you fill out the tax claim” (Kim, Austria, 1. Round)* as one explained. Caregivers felt overwhelmed by the instructions and often misunderstood them. As participants often missed the four-month time frame to answer, it was mentioned in YC-PEM (G) on each page. Eighty-two percent of all operational adaptations were made in the instructions. The one-page introduction in YC-PEM was divided into two pages in YC-PEM (G); part 1 was for the three participation settings and part 2 was for the adherent environment sections.

**Figure 2:**
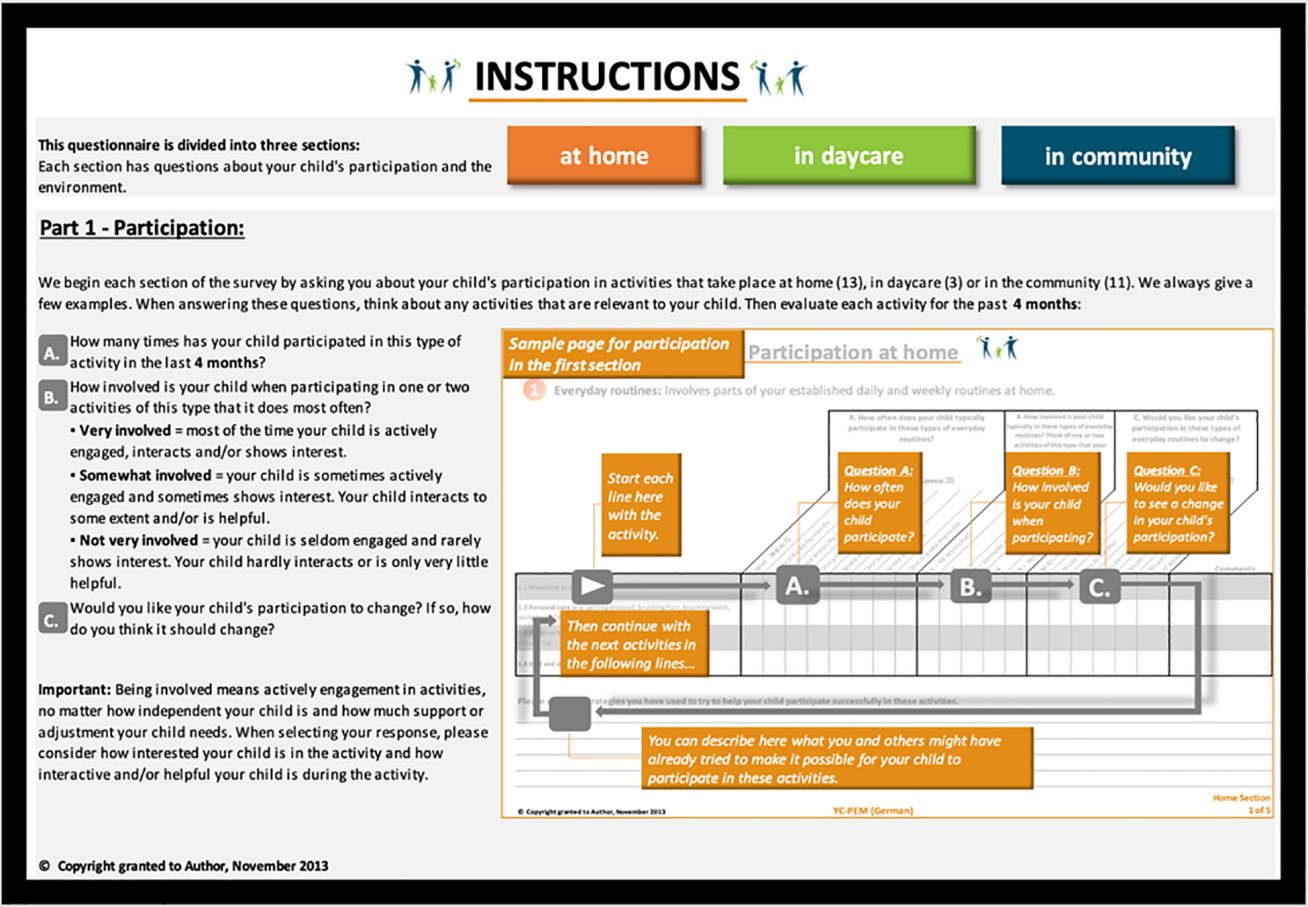
Page two YC-PEM (G) instructions (back translation) participation.

**Figure 3:**
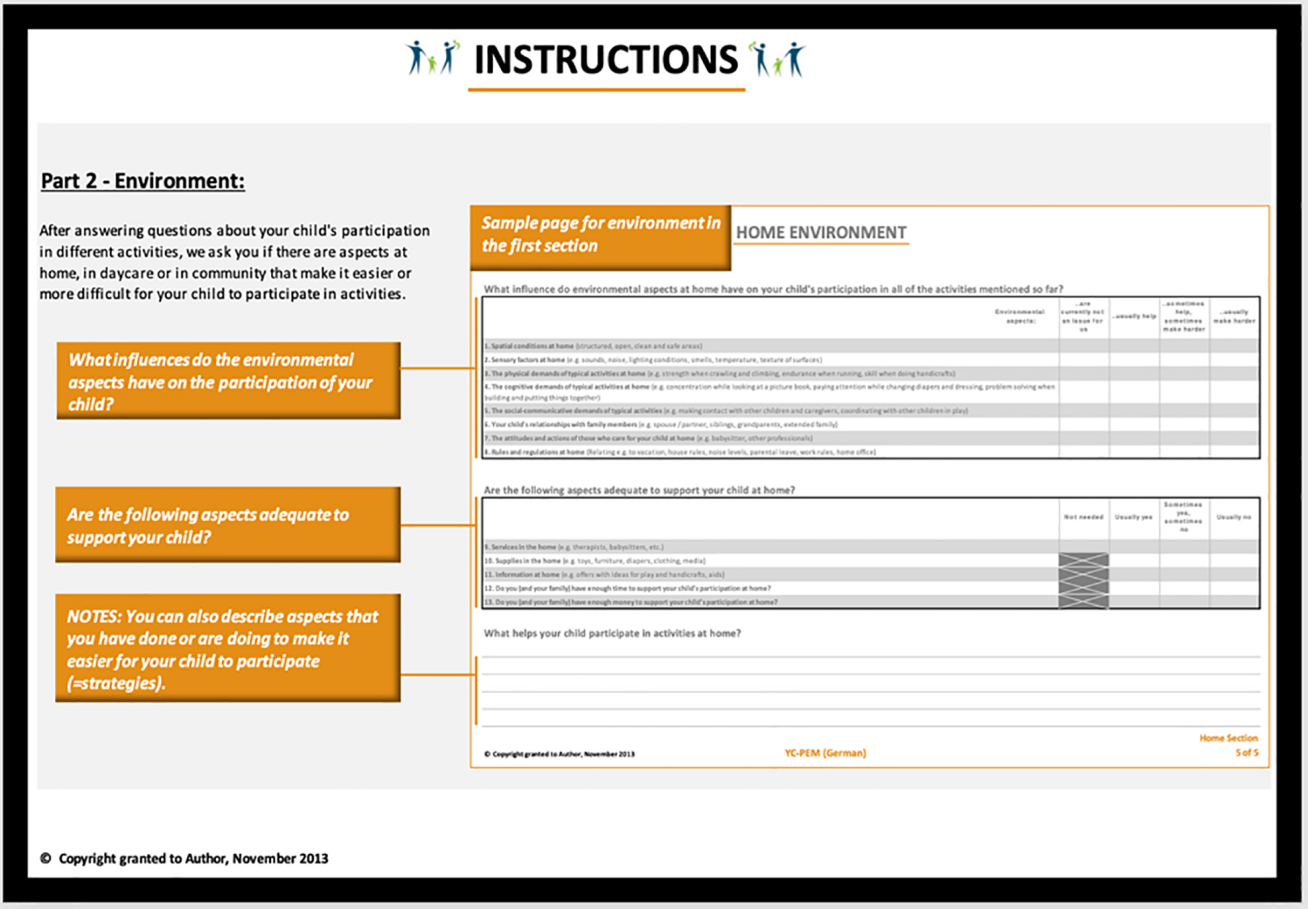
Page two YC-PEM (G) instructions (back translations) environment.

Furthermore, the instructions in the 2nd round were changed. While the verbal instructions were simplified, the supplemented graphics indicated the process of filling out the YC-PEM (G): we observed that caregivers often oriented themselves vertically, while with the graphics, a correct horizontal procedure becomes obvious. The researchers presented different graphical versions in the second interview so that caregivers could indicate the most convenient one. Additionally, to give orientation for the three settings of YC-PEM (G)—home—daycare/preschool—community, the colors used in the YC-PEM were used more prominently in YC-PEM (G). During the last round of interviews, participants refrained from commenting negatively on the operational modes of the assessment. Figure 2 shows a visual example of the participation instruction in YC-PEM (G), while Figure 3 shows the instruction for the environment section. Both examples *are excerpts from the English back translations to* inform the developers.

Lastly, for caregivers who did not use any daycare facilities for their children, starting to answer the daycare/preschool section of YC-PEM was irritating. Consequently, in YC-PEM (G), a prominently placed question asks caregivers whether their children had attended any daycare facilities during the last 4 months. If not, YC-PEM (G) instructs to skip this second section.

### 3.5 Conceptual equivalence

The fact that we could build on the main concepts of the cultural adaptation of PEM-CY into German reduced adjustments to conceptual equivalence significantly. However, some conceptual issues still need consideration.

Firstly, we heard in the interviews with caregivers that participation frequency is a concept that is not always important to caregivers of young children. “*Sleep and rest, mealtime, and daycare are activities all young children participate in each day.* So, I don't understand why frequency is important. I think the quality is much more important; how does she eat? Does she eat the healthy stuff we offer?” (Naomie, Switzerland, 1. Round). The research team could not address this problem as it seems to touch the very core of the assessment. However, by filling in additional space after each activity in YC-PEM (G) for comments, parents could at least explain their thoughts and judgments.

Secondly, “involvement” is a concept that caregivers of young children give parents a lot to think about. Consequently, it was not easy to fill in. As there is no single word to translate «involvement» in German, we used engagement (G: Engagement) and explained it as being active, supportive, and interactive. Two caregivers of small children under the age of one expressed how they observe this engagement when their children are curious and take an interest in something, even when they cannot yet be active or supportive. The second round of interviews tested “interest and curiosity”, and parents reacted overly positively to this explanation. However, after the final backtranslation, there was an intensive conversation with the developer team from CanChild about the understanding of “involvement.” While caregivers from this present sample rated strongly whether their young children showed interest and curiosity, parents of the original sample of the developers (20) rated strongly how actively children contributed to an activity. In an exchange with the developers, we discussed whether this was due to different cultural imprints, convictions, and socialization goals, such as an individualistic vs. a collectivistic view. Probably, parents from Western European cultural areas differ from North American or South Asian parents; thus, their reflection of their belief systems is specific to their context. This aspect should be considered in future research and cultural adaptation of the measurement. At the end of this iterative discussion, the research team decided to keep “interest” in the explanation of “involvement” in the YC-PEM (G). A caregiver of a young autistic boy explained how she thinks it is important to keep various observations in “involvement”: “*My non-verbal boy can interact with me about a toy train, or he can support me in putting the toy locomotive on the tracks. I think both present a form of engagement, and I value that both are included here*” (Sarah, Switzerland, 1. round). The importance of including various aspects in “involvement” is also reflected in the comment of another mother: “*When it comes to shopping, she can’t get actively engaged because there she is in the stroller, she would clean everything out” [Sic] (Mary, Austria, 2.round).*

Thirdly, caregivers often asked themselves during think-aloud interviews how independently their children performed activities. Some wondered how their young children should prepare meals or participate in their own meetings. So, we needed to express that independence was not a concept asked for in YC-PEM (G).

Fourthly, YC-PEM asked caregivers to name strategies and when they wished for changes in participation frequency and involvement. The research and expert teams deliberately wanted to strengthen this part by asking caregivers about strategy even when they did not wish for any changes. Participants overly accepted this; hence, one mother of a healthy young girl said: “*There is no need for parental strategies. She's curious; that's enough.” (Naomie, Switzerland, 1.round).*

Fifthly, caregivers found it less suitable and sometimes even impossible that in the environments section, global environments (such as the physical layout in the community) are judged commonly: “*I think it's less meaningful that way because you can't relate it to a specific situation”, (Jane, Germany, 2. round)* while another caregiver explained: “*Yes, in the grandparents’ house she can move more freely, she can go into the garden, while in in the hotel in Italy, she can't go to the beach because there is a street in between” [sic] (Julia, Austria, 1. round).* By inserting the words “aspects of the environment”, YC-PEM (G) tries to help parents to take a step back and evaluate aspects of the environment from a broader stance.

Lastly, one caregiver criticized the fact that children cared for partly by grandparents are not mentioned. However, as we have included the foster care situation and several words and conditions for external daycare, we have decided to refrain from inserting “grandparents” as a particular daycare situation.

### 3.6 Measurement equivalence

As no adaptions in psychometric properties were performed for YC-PEM (G), we have no data to assure measurement equivalence.

### 3.7 Cultural equivalence

When cultural equivalence is seen as a combination of the previously mentioned six equivalences, the authors are convinced that this study succeeded to a large extent. The overall feedback from participants to the questionnaire was constructive and positive. A mother of a child without disability stated: “*It has only now become clear to me how privileged we would live. We have an optimal environment, and I am totally happy how my child can participate in it.”* [sic] (Sofia, Switzerland, 1. round). Parents of children with a disability often needed more time to reflect on their children's participation and how they could summarize the different environmental conditions. A mother of a boy with autism spectrum disorder reflected: “*I have been asked for pages how my child handles a ball. I found this one very useful. It also opened my eyes to what is really important for me with my child.”* (Sarah, Switzerland, 1 round).

## 4 Discussion

This study aimed to cross-culturally translate and adapt the YC-PEM into German as it is used in Germany, Austria, and Switzerland. A collaboration between researchers, experts, caregivers, developers, and linguists reached cultural equivalence with changes predominantly considering item, operational, and conceptual equivalence. The operational changes also enhanced the user-friendliness of the German version YC-PEM (G). We are going to discuss these three most relevant equivalence aspects further (item, operational, and conceptual).

Firstly, referring to item equivalence (41), it was expected that activity and participation-based assessments needed item adaptation when transferred to other cultures, as activities and participation represent concrete artifacts of culture. How the YC-PEM was constructed (the exemplary naming of the most frequently typical activities of a cultural community) increased the number of changes to reach item equivalence additionally. Sometimes also defined as content equivalence (39), it was reported to be also high in similar research (30, 33, 34, 50). We also agree with Arestad et al. (34) that some items of YC-PEM were less relevant to children of a younger age, and this became obvious in think-aloud interviews of caregivers with infants. Adapted to the age frame, items such as “baby sport”, “slagging”, and “climbing” were more present in the German version (due to the added examples in the environment parts). Interestingly, we made numerous item changes in unstructured community activities: outdoor activities such as “playing in outdoor playgrounds, parks, forest, on the beach, moving, swimming, scooter/cycling, and tobogganing” were of higher relevance in German-speaking contexts. A possible explanation is that inner city centers are much smaller in our countries than in North America.

Secondly, operational equivalence has gained, until now, less focus on established equivalence guidelines (39). However, it is with aesthetics, layout, instructions, and administration important to (1) ensure that caregivers continue to fill out the questionnaire and (2) frame the questions correctly (35). Although it is mentioned in all culturally adapted versions of YC-PEM (30–34), it is, to the best of our knowledge, the first cultural adaption to change the instructions of YC-PEM graphically, with results, and that caregivers in the interviews did not complain about the seemingly long instruction. However, others describe a similar approach to visualizations after the caregivers’ feedback in their first round of cognitive interviews with the PEM-CY (51). With this adapted introduction and the use of colors as guiding means (52), changes in the display, which are reported frequently elsewhere (33, 34), are less needed. These adaptations also increase the self-explanatory nature of the YC-PEM and support the concept of self-report assessments. Users should be able to complete an assessment without the help of professionals. User-friendliness is a vital topic in assessment construction (53) and combines with “user literacy” in health service delivery (54). From a participatory point of view, the methodical involvement of users as researchers, experts, or think-aloud testers for the process of cultural adaptations is central. Recently published PEM guidelines (35) recommend performing various rounds of interviews and with a selected, diverse sample with a range of experience. As professionals are used for language reading and assessments, we prefer users instead of professionals for think-aloud interviews. However, without encouraging and supportive developers, these visual adaptations could not be implemented in the assessment.

Thirdly, conceptual equivalence results in this present study are more important than previously expected. By referring to experiences of the cultural adaptation of PEM-CY for the German-speaking countries (43) and thus integrating the primary conceptual phrasing from PEM-CY (G) into YC-PEM (G), we decided that sufficient information in an early stage of the project would prevent problems later on (41). This was definitely the case considering the wording of “participation”, “environment”, and “community”, which in the Dutch cultural adaptation posed some difficulties (33). Similar to findings by others (31, 34, 51), expert consultation in an earlier stage led to even more conceptual equivalence changes. However, think-aloud interviews not only deliver a keen understanding of assessments but also question the concept of an assessment as a whole. In our case, these were (1) how to define “involvement” in young children and (2) what to do if parents do not estimate participation but other qualities for their children as more desirable (such as independence, obedience, creativity, and self-expression). These findings were different from previous cross-cultural adaptation studies of YC-PEM that did not find any adaptation needs on a conceptual level (35). Adaptations on a conceptual level could be discussed by understanding the participation construct in relation to other constructs as described by Imms et al. (2017) in the family of participation-related constructs (3). The family of participation-related constructs and intrinsic-related elements was described as elements that “are both antecedents of future participation and consequences of current or past participation” (4). In this current cross-cultural adaptation, caregivers seem to have related concepts, such as independence, in mind while taking the YC-PEM. Such related concepts might grasp their child's past, present, or future participation. Even when a caregiver's perspective might be tainted with related constructs, a cross-cultural adaptation process should ensure that conceptual equivalence is achieved as these are the core constructs the YC-PEM is measuring. An avenue of future interpretation would be to provide additional evidence in the form of psychometric property studies within the German language regions to confirm the conceptual equivalence.

Methodologically, this research was oriented along guidelines for participation-focused assessments in childhood (35), adapted the processes described by Beaton et al. (40) iteratively, and referred to definitions of equivalence by Herdman, Fox-Rushby, and Badia (29) modified to participation assessments by Stevelink and Brakel (41). This allowed a flexible, iterative process and included operational equivalence, which became immensely important to reach cultural equivalence of the YC-PEM for German-speaking countries [YC-PEM (G)]. Secondly, integrating an expert panel with various child-specific health professionals from all three countries and caregivers of young children allowed this assessment to be also used interprofessional. Thirdly, the combination of qualitative comments obtained from both the expert committee and caregiver interviews, along with the quantitative descriptive outcomes from the summative content analysis in this study, proved to be valuable. For example, during the adaptation process, caregivers expressed a need for revisions in the introduction and explanation of the measurement instrument. This led to specific operational improvements of the YC-PEM that might not have been identified if caregivers had not pointed out challenges in comprehending this section during the think-aloud interviews. We urge that such adaptations are crucial as these will support future reliability testing of the YC-PEM German. The objectivity and reliability of the procedure would thus be limited.

The strength of this study lies in a methodological, careful, multi-perspective process of cultural adaptation into German as it is spoken in three countries. It integrates previous cultural adaptions of similar instruments (43) and contributes to a consistent use of participation-focused language in German.

We see five limitations of this work. Firstly, we have slightly fewer think-aloud interviews as recommended (35) due to Corona lockdowns and project time limitations. This might result in a too-optimistic judgment of equivalence. Secondly, although we were looking for a diverse sample of caregivers with children with and without disabilities, different social class privileges, and different countries, we cannot assure that persons with a minor health literacy or reduced language competencies can understand this assessment. Thirdly, we did not address measurement equivalence because no adaptations were made in the measurement. However, it could be argued that until it is not tested, a measurement equivalence is not reached (41). Fourthly, a limiting factor in utilizing the YC-PEM could be the time required for refilling. This could be challenging in everyday clinical practice. However, it should be considered that this result is a very intensive, structured exchange of information, which facilitates rehabilitation planning and enables a fit with the individual life situation. It also remains to be seen how the YC-PEM, which is similar to the PEM+, could be transferred into a shorter digital form (55). Lastly, as we were pilot testing the pre-final version of the YC-PEM (G) face-to-face, how users would understand an online version in which they have to rely 100% on written instructions cannot be assured.

Next, the YC-PEM (G) must be applied and tested to establish psychometric properties, reliability, and validity for different groups of young children and practice contexts. It also needs to be disseminated in German-speaking contexts.

## 5 Conclusion

This study presents a cross-cultural translation and adaptation process to develop a German version of the YC-PEM that is suitable for three German-speaking countries. As participation and environment are both complex concepts to measure, item equivalence and conceptual equivalence posed the greatest challenges for this cultural adaptation. The adaptation uses new graphical instructions to ease understanding and processes to fill in the assessment correctly. With extensive input from parents, experts, researchers, and the YC-PEM team from CanChild, a culturally adapted version of YC-PEM (German) is now available for research, practice, and further validation.

## Data Availability

The original contributions presented in the study are included in the article/Supplementary Material, further inquiries can be directed to the corresponding author.
